# Oral hygiene practice and periodontal status among two tribal population of Telangana state, India- an epidemiological study

**DOI:** 10.1186/s12903-018-0705-1

**Published:** 2019-01-09

**Authors:** Shaik Mohammed Asif, Shaik Naheeda, Khalil Ibrahim Assiri, Hussain Mohammed Almubarak, Sultan Mohammed Kaleem, M. Zakirulla, Fawaz Abdul Hamid Baig, Mohammed Zahir Kota

**Affiliations:** 1 Department of Diagnostic Science & Oral Biology, College of Dentistry, King Khalid University, Abha, Kingdom of Saudi Arabia; 2 Department of Periodontology, Guardian College of Dental Science and Research Centre, Ambernath, Thane, India; 3 Department of Diagnostic Sciences & Oral Biology, College of Dentistry, King Khalid University, Abha, Kingdom of Saudi Arabia; 4 Department of Pediatric Dentistry & Orthodontic Sciences, College of Dentistry, King Khalid University, Abha, Kingdom of Saudi Arabia; 5 Department of Maxillofacial Diagnostic science, College of Dentistry, King Khalid University, Abha, Kingdom of Saudi Arabia

**Keywords:** Tribes, Periodontal disease, Community periodontal index, Oral hygiene index- simplified

## Abstract

**Background:**

The tribes of India have poor periodontal health status due to their isolation, awareness and less accessibility to dental resources. They follow traditional methods of oral hygiene practice, which are found to be inadequate and inaccurate to maintain their good oral health. This study aims to assess the oral hygiene practice, oral hygiene and periodontal status of two tribes residing in Bhadrachalam, Telangana, India.

**Methods:**

Based on accessibility the two tribes Koya and Lambada were included in the study. The total study population consisted of 1000 subjects, with 500 subjects in each group. Using a preformed proforma the oral hygiene practices were recorded for two groups and oral examination was done using Oral Hygiene Index (OHI-S) index and Community Periodontal Index (CPI).

**Results:**

The OHI-S ranged between 2.5–3.0 in both the groups. The CPI index scores showed that sextants with calculus, shallow pockets, deep pockets and loss of attachment of 4-5 mm were significantly present in both groups.

**Conclusion:**

To conclude, though practice of poor oral hygiene and compromised periodontal status was seen among both groups. However, it was more prevalent in Koya. Under these circumstances, implementation of a basic awareness of oral health care programme for these tribes should be a high priority.

## Background

Various research have designated that, periodontal disease are as ancient as mankind. It could be due to difference in life style and oral hygiene habits [1]. Though efforts have been put fighting against periodontal disease, problem still persist among poor and socially marginalized communities. The situation is particularly severe in developing countries like India [2]. In this modern era, there are people who are living in isolation in natural and unpolluted surrounding far away from civilization with their traditional values, customs, beliefs and myths. They are known as “tribes,” for which India is a homeland providing shelter for 75 of such tribal communities [2]. The primitive tribal communities have been identified by government of India in 15 states/union territories on basis of pre agricultural level of technology, extremely low level of literacy; and small stagnant or diminishing population [3]. The only south Indian state with tribal population of 9.34% is Telangana out of its total population as per 2011 census [3]. However, most of these tribes are found inhabiting in higher concentration in districts of north and northeastern parts of State. In Telangana, Khammam district has largest tribal population. Koyas and Lambadas are the major tribes leaving in the ITDA (Integrated Tribal Development Agency) of Bhadrachalam [4]. Koyas due to their low annual income and lack of land ownership they work as daily farm laborers [5]. Lambadas live in settlements referred as Thanda [5, 6]. They are more civilized when compared to Koyas [6]. Research has shown that low socioeconomic and ethnic minority groups are less likely to utilize health service [7]. Hence, evaluation of oral health in these communities is an important part for deciding accurate and respectable health care. As there is no literature available, regarding oral health status of these population, this present study aimed to assess the oral hygiene practice and periodontal status of these two tribes inhabiting in Bhadrachalam division of Khammam district, Telangana, India.

## Methods

It is a cross sectional epidemiological study conducted to assess the oral hygiene practice and periodontal status of two tribes residing in Bhadrachalam, Telangana, India. It has been systemically scheduled to spread over a period of two and half years (July 2015 to January 2018). Institutional review board of private dental college issued an ethical clearance and ITDA Officer of that area permitted to conduct the study. With guidance from ITDA office and help from dental auxiliaries of that region, it was made sure that eloquent number of samples were accessible during the day of examination. An informed consent verbally and a written declaration were obtained from participants after discussing in detail about the purpose of the study.

### Sample size collection

A preliminary study was conducted to determine the sample size on the results obtained and to check for the feasibility of participants during the study. After conducting the pilot study the prevalence of periodontal disease was assessed and the sample size attained as follows: The prevalence of periodontal disease in I tribal group P1 = 0.45% (Proportion in the I group P1 = 0.4520) The prevalence of periodontal disease in II tribal group P2 = 0.56% (Proportion in the II group P2 = 0.5656) Risk difference (P1-P2) = − 0.1136 Power of test (%) = 95 Alpha Error(%) = 5 n = [(Zα/2 + Zβ) 2 × {(p1 (1-p1) + (p2 (1-p2))}]/(p1 - p2)2 p1 = Proportion in the first population p2 = Proportion in the second population α = Significance level β = Power of test Zα = (1.96) Table value Zβ = (1.682) Table value Sample size *n* = 500 in each group. Hence, total sample size was thousand. We used a modified WHO proforma which had queries related to oral hygiene practice that might have indirectly affected on Periodontium and its supporting structures [8]. To ascertain the prevalence of periodontal disease and the attachment loss CPI index was used (Jukka Ainamo, David Barmes, George Beagrie (1994) [8].

### Inclusion criteria

Subjects willing to participate in the age group of (20 to 75 years), and those who were present during the day of examination were included in the study.

### Exclusion criteria

Children, edentulous patients and those subjects who were absent or not willing to participate in the study were excluded.

Oral examination was conducted in regional hospital of Bhadrachalam Mandal for the Koya group and Pichakalapadu Village for the Lambada group. Intra- examiner variability, was reviewed by department staff of Periodontics, private dental College to reduce intra-examiner variability before conducting the pilot study. Autoclave method was used to sterilize the instruments in sterilization section of the area hospital, Bhadrachalam. If needed chemical sterilizer was used before oral examination. For collection of data, a specially designed pre tested proforma was used. The proforma consists of three parts. The first part was questions related to demographic data such as age, gender, occupation and address. The second part of questions were related to oral hygiene practice. The third part of the questionnaire was clinical examination of the subjects using OHI-S and CPI indices (Greene & Vermillion − 1964) [8, 9]. The observed results were analyzed using Statistical Package for Social Science (SPSS 20) by IBM. Chi- Square test was used to determine the distribution of study samples by type of tribal population and various parameters such as age, gender, occupation, oral hygiene practices. Kruskal Wallis Anova and Mann- Whitney U test was used to compare the two tribal population with respect to OHI-S index and CPI index. *P* < 0.05 was considered statistically significant.

## Results

The study population was of 1000, with 500 subjects from each group. As per results of present study, the mean age of subjects in Koya and Lambada groups was 40.52 ± 14.69 and 39.34 ± 13.77 years. Majority of participants were females (Table.1). A statistically significant difference (*p* < 0.05) was observed with respect to occupation among these two tribes. In Koya group most of the participants were farmers (69%) and mainly cultivated tobacco 23.4% were daily labors (Table 2). The occupation of Lambada group was business (65%) followed by daily wage labors and farmers (Table 2). In Koya group 37.40% used twigs to clean their teeth, 32.6% used tooth brush and 17.6% used finger to clean their teeth (Table 3). However, in Lambada group 46.4% used tooth brush, 30% used twig and only 9.4% used finger to clean their teeth (Table 3). In Koya 36.8% did not use any material for cleaning their teeth and only 22% used tooth powder for cleaning their teeth (Table 4). In Lambada group 30.8% used tooth paste, 29.4% did not use any material and 25.8% used sand and others as material for cleaning their teeth (Table 4). In Koya group, 64% of them were not sure of using any particular type of toothbrush ands only 13.6% of them were using soft tooth brush. 73.2% of Koya group and 74% of Lambada group cleaned their teeth in horizontal direction followed by a combination of horizontal and vertical method (Table 5). All these participants brushed their teeth once daily and mostly before meals in the early morning. A significant difference was observed with frequencies of change of brush in two tribal group (*p* < 0.05) 63% of Koya group and 51.2% of Lambada group never changed their brush. Only 31.80% of participants of Koya group changed their brush once in 3 months. Where as in Lambada group, 46.00% of them changed their brush once in 3 months (Table 6). 51.2% of Koya group and 63.4% of Lambada group rinse their mouth after every meal (Table 6). The mean Debris Index simplified (D.I) were 1.20 ± 0.43 for Lambada and 1.24 ± 0.42 for Koya group which was statistically significant (Table 7). The mean calculus Index simplified (C.I) were 1.33 ± 0.61 for Lambada and 1.35 ± 0.49 for the Lambada group which was statistically significant (Table 7). The mean OHI-S index values for Koya and Lambada group were 2.56 ± 0.82 and 2.51 ± 0.93 (Table 7). The mean number of sextants affected by periodontal disease according to CPI Index in Koya group for bleeding on probing was 0.51 ± 0.81 and in Lambada group it was 0.42 ± 0.68 which was statistically significant (Table 8). The mean calculus in Koya group was 4.11 ± 1.46 and in Lambada group it was 4.37 ± 1.29 which was statistically significant (Table 8). Among periodontal conditions, calculus was more prevalent in both groups followed by shallow pockets i.e., Pockets measuring 4-5 mm. (Table 8). In comparison of two tribal population for mean number of sextants affected with periodontal disease according to loss of attachment component of CPI Index (Table 9) shows that, very few sextants in both groups showed loss of attachment of ≥6 mm. None of the participants in either of the group showed loss of attachment ≥12 mm. (Table 9).

**Table 1:** Distribution of gender of tribal population

Gender	Koya	%	Lambada	%
Male	215	43.00	222	44.40
Female	285	57.00	278	55.60
Total	500	100.00	500	100.00

Chi-square = 0.4270, df = 2, *p* = 0.8076

**Table 2:** Distribution of Occupation of Both Tribes

Occupation	Koya	%	Lambada	%
Agriculture	345	69.00	52	10.40
Business	10	2.00	325	65.00
Coolie	117	23.40	94	18.80
Housewife	28	5.60	12	2.40
Others	0	0.00	17	3.40
Total	500	100.00	500	100.00

Chi-square = 293.5892, df = 8, *p* = 0.00001*

*P < 0.05

**Table 3:** Distribution of type of cleaning aid used among both tribes

Type of cleaning	Koya	%	Lambada	%
Tooth brush	163	32.60	232	46.40
Finger	88	17.60	47	9.40
Twig	187	37.40	150	30.00
Combinations	62	12.40	71	14.20
Total	500	100.00	500	100.00

Chi-square = 540.6722, df = 6, *p* = 0.00001*

*P < 0.05

**Table 4:** Distribution of Materials used for cleaning by both tribes

Materials used for cleaning	Koya	%	Lambada	%
None	184	36.80	147	29.40
Tooth Paste	110	22.00	154	30.80
Tooth Powder	5	1.00	0	0.00
Charcoal	66	13.20	74	14.80
Sand and others	135	27.00	125	25.00
Total	500	100.00	500	100.0

Chi-square = 496.642, df = 8, *p* = 0.00001*

*P < 0.05

**Table 5:** Distribution of Method of cleaning among both tribes

Methods of cleaning	Koya	%	Lambada	%
Vertical	17	3.40	10	2.00
Horizontal	366	73.20	370	74.00
Circular	1	0.20	0	0.00
Vertical and Horizontal	77	15.40	100	20.00
Horizontal and Circular	39	7.80	20	4.00
Total	500	100.00	500	100.00

Chi-square = 81.5732, df = 8, *p* = 0.00001*

*P < 0.05

**Table 6:** Distribution of frequency of Brushing, Frequency of Change of brush, and Rinsing of mouth after meals by both tribes

Frequency of brushing	Koya	%	Lambada	%	Frequency of change of brush	Koya	%	Lambada	%	Mouth rinse after Meals	Koya	%	Lambada	%
Once	500	100.	500	100.0	None	315	63.00	256	51.2	Yes	256	51.20	317	63.40
Twice	0	0.0	0	0.0	1 month	5	1.0	2	0.4	No	244	48.80	183	36.60
Total	500	100.	500	100.0	3 months	159	31.8	230	46.	Total	500	100.0	500	100.0
					4 months	4	0.8	0	0	Chi-square = 16.0060			df = 2	*p* = 0.00034*
					6 months	17	3.4	12	2.4					
					Total	500	100	500	100					
					Chi-square = 333.9526		df = 8	*p* = 0.00001*						

*p < 0.05

**Table 7:** Minimum, maximum, mean and SD of Debris index, calculus and OHIS scores of two tribal populations

Tribes	Debris Index (DI)			Calculus Index (CI)			OHI_S		
	Mean	SD	Sum of ranks	Mean	SD	Sum of ranks	Mean	SD	Sum of ranks
Lambada	1.20	0.43	334,815	1.33	0.61	337,490	2.51	0.93	334,915
Koya	1.24	0.42	352,120	1.35	0.49	349,945	2.56	0.82	347,625
*P*-value		0.00001*			0.00001*			0.00001*	
Pair wise comparison of both tribal populations by Mann-Whitney U test									
Koya vs Lambada		0.1903			0.2724			0.2545	

*p < 0.05 (Kruskal Wallis ANOVA and Mann-Whiney U test)

**Table 8:** Comparison of two tribal population for - Mean number of sextants affected by periodontal disease condition according to CPI index

Variables		Tribes		H-value	*P*-value	Pair wise comparison		
		Koya	Lambada			Koya vs Lambada		
Healthy	Mean	0.57	0.49	150.17	0.00001*			0.35
	SD	0.98	0.89					
Bleeding	Mean	0.51	0.42	54.89	0.00001*			0.29
	SD	0.81	0.68					
Calculus	Mean	4.11	4.37	43.69	0.00001*			0.015
	SD	1.46	1.29					
Pocket (4–5 mm)	Mean	0.51	0.58	73.85	0.00001*			0.090
	SD	0.82	0.83					
Pocket (6 mm or more)	Mean	0.01	0.01	2.84	0.2420			0.96
	SD	0.09	0.08					
Excluded sextants	Mean	0.01	0.01	2.43	0.2130			0.87
	SD	0.08	0.08					
Total	Mean	1.74	1.82	134.69	0.00001*			0.01*
	SD	0.48	0.44					

*p < 0.05 by Kruskal Wallis ANOVA and Mann-Whiney U test

**Table 9:** Comparison of two tribal populations for - Mean number of sextants affected with periodontal disease according to Loss of Attachment component of CPI index

Variables		Tribes		H-value	*P*-value	Pair wise comparison
		Koya	Lambada			Koya vs Lambada
Score − 0	Mean	4.03	3.83	57.3970	0.00001*	0.0812
	SD	1.95	1.89			
Score − 1	Mean	1.37	1.75	86.6340	0.00001*	0.0003*
	SD	1.49	1.55			
Score − 2	Mean	0.29	0.27	7.7850	0.0200*	0.9999
	SD	0.74	0.70			
Score − 3	Mean	0.05	0.01	10.7910	0.0050*	0.5460
	SD	0.31	0.14			
Score − 4	Mean	0.00	0.00	0.0000	1.0000	1.0000
	SD	0.00	0.00			
Excluded sextants	Mean	0.01	0.01	2.43	0.2130	0.87
	SD	0.08	0.08			
Total	Mean	0.38	0.40	56.6280	0.00001*	0.0537
	SD	0.46	0.40			

*p < 0.05 by Kruskal Wallis ANOVA and Mann-Whiney U test

## Discussion

This study aims to assess the oral hygiene practice, oral hygiene and periodontal status of two tribes residing in Bhadrachalam, Telangana, India. Primitive tribal communities have been identified by the Government of India based on their pre agricultural level of technology, extremely low level of literacy and small, stagnant or diminishing population [3, 7]. Major participants in the study were females. Similar findings were noticed among Nilgiris [1] tribes where participation of females (58%) was more compared to males. This could be attributed to their esthetic concerned and co-operative nature of females [1]. The occupation of major participants in Lambada group was Business (65%) followed by daily wage laborers and farmers as they are more civilized and have better communication with modern society through transport and most of them were into business as their occupation. Majority of Koyas used twig as their regular oral hygiene method and Lambadas used tooth brush as their cleansing aid. Similar findings were noticed by Jordan RA [10] in rural population of Gambia in West Africa, where majority of the population used chew sticks (50.6%) followed by toothbrush (34.6%). Similarly Padma BK et al. [11] reported that chew sticks were commonly used than toothbrushes as an oral hygiene aid among Iruliga tribes in Karnataka, India. Contrastingly, Khadir et al. [12] who conducted a study in aborigines of Selangor, West Malaysia reported that majority of the population used toothbrush with toothpaste and they brushed their teeth once daily. The authors suggested that these responses from the participants could have been influenced by factors such as desirability of participants to answer questions on dental health and behavior in a socially desirable way [12]. The mean OHI-S index [9] values for Koya and Lambada indicated a fair oral hygiene status. The present findings are in accordance with similar studies conducted on other communities like Australian Aborigines [13], residents of Kolar district [14] and municipal employees of Mysore [15]. The high mean values of the oral hygiene index and its components suggest a widespread and uniform neglect of tooth cleaning/ brushing habit among tribal groups [1]. The average number of sextants with healthy condition in present study and sextants with Bleeding on probing were significantly less in Koya and Lambada groups. Similar findings were noticed by Jordan et al. [10] in rural African Gambia population. The authors proposed that use of two tooth cleaning aids i.e., twigs and toothbrush lead to decrease gingival inflammation [10]. Sextants with calculus, shallow pockets and deep pockets were high in Koya and Lambada groups. Among all the periodontal condition, Koya’s had least calculus when compared to Lambadas. This is due to the fact that, the people belonging to this tribe consume large amounts of vegetables, tubers and roots, which may have a self-cleansing effect on the teeth [16]. The diet also plays a role in maintaining their oral hygiene as the diet of Koya tribe includes highly abrasive food while excluding those consisting mostly of refined carbohydrates [16]. Periodontitis of any form was less prevalent in Lambada tribe it is due to their awareness about oral health owing to their communication with modern society for the purpose of trade.

## Conclusion

Lambadas had better oral hygiene practice. Poor oral hygiene status and periodontitis was more prevalent among Koyas which could be related to their lack of knowledge regarding the dental care. There is a consensus agreement that periodontal health status of tribal population is very poor and worst among primitive tribes because of their isolation, remoteness therefore, dental resources should be made easily accessible. Ethnic believes which inhibit the use of health care services can be overcome by motivating, training primary health care workers who are in continuous contact with these tribes. This can help bridge the gap between oral health practice and oral health needs of tribes. Hence, community based approach for promotion of good oral hygiene should be carried out among these tribes on a large scale for control and prevention periodontal disease.

## Abbreviations

C.I: Calculus Index Simplified
CPI: Community Periodontal Index
D.I: Debris Index Simplified
ITDA: Integrated Tribal Development Agency
OHI-S index: Oral Hygiene Index
WHO: World Health Organization
